# Human Protein Cluster Analysis Using Amino Acid Frequencies

**DOI:** 10.1371/journal.pone.0060220

**Published:** 2013-04-04

**Authors:** Annamaria Vernone, Paola Berchialla, Gianpiero Pescarmona

**Affiliations:** 1 Department of Oncology, University of Torino, Orbassano (Torino), Italy; 2 Department of Clinical and Biological Sciences, University of Torino, Orbassano (Torino), Italy; George Washington University, United States of America

## Abstract

The paper focuses on the development of a software tool for protein clustering according to their amino acid content. All known human proteins were clustered according to the relative frequencies of their amino acids starting from the UniProtKB/Swiss-Prot reference database and making use of hierarchical cluster analysis. Results were compared to those based on sequence similarities. Results: Proteins display different clustering patterns according to type. Many extracellular proteins with highly specific and repetitive sequences (keratins, collagens etc.) cluster clearly confirming the accuracy of the clustering method. In our case clustering by sequence and amino acid content overlaps. Proteins with a more complex structure with multiple domains (catalytic, extracellular, transmembrane etc.), even if classified very similar according to sequence similarity and function (aquaporins, cadherins, steroid 5-alpha reductase etc.) showed different clustering according to amino acid content. Availability of essential amino acids according to local conditions (starvation, low or high oxygen, cell cycle phase etc.) may be a limiting factor in protein synthesis, whatever the mRNA level. This type of protein clustering may therefore prove a valuable tool in identifying so far unknown metabolic connections and constraints.

## Introduction

“Epigenetics” can be broadly used to describe any aspect other than a DNA sequence able to alter a phenotype without changing its genotype. The science of epigenetics – the study of reversible changes in gene function that occur without a change in the DNA sequence – is transforming the nature-nurture debate. It has been speculated that dynamic epigenetic processes, operating at the interface between the genome (nature) and the environment (nurture), strongly influence the complexity of living organisms in health and illness [1]. Cell chemical processes used to regulate gene expression and specific mRNA synthesis (transcription) include methylation, phosphorylation, acetylation and are usually regarded as canonical tools of this regulation. However, the step from mRNA to protein (translation) also displays absolute requirements including ribosomal machinery, tRNA, ATP supply and amino acids (AA) local availability.

To date the effect of AA availability as regulating factor of every protein synthesis has not been extensively investigated. It is well known the glutamine requirement for purine bases synthesis [2] or the leucine effect on mTOR expression [3], but usually protein synthesis rate is correlated with mRNA amount and not with local essential AA concentration.

In our research we assume that local AA availability is a limiting factor for a given any protein synthesis. It is well known that mRNA has a limited life span and that factors affecting its expression and stability are powerful modulators of protein synthesis. In the case of AA scarcity the rate of some tRNA-aminoacid complexes may become the prevailing limiting factor, provided the mRNA lifespan is shorter than the time required to collect all the required AA.

We therefore hypothesize that the relative abundance of proteins in different cellular setup may also depend on the local availability of AA. The AA percentage of a protein should mirror AA local availability. Our clustering tool is intended for identification of homogeneous groups of proteins whose synthesis can be regulated by selected AA relative abundance in proper experimental settings.

The protein sequences of all human genes were extracted from the reference database UniProtKB/Swiss-Prot and clustered using agglomerative, or bottom-up, hierarchical cluster analysis. Every protein initially corresponds to one-point cluster and, in each subsequent step, the two ‘closest’ clusters were merged until only one remained. The agglomerative approach offered advantages such as more ﬂexible clustering as well as often producing higher quality trees.

## Materials and Methods

### Data

The source of data: the protein sequence in FLAT file format from UniProtKB/Swiss-Prot protein database, which provides protein sequences with extensive annotation and cross references. The database is regularly updated and is a section of UniProtKB [4]. UniProtKB is organized in two sections:

1. UniProtKB/Swiss-Prot, which is the main database, manually curated, which means that the information in each entry is annotated and reviewed by a curator;
2. UniProtKB/TrEMBL, which is the supplement database of Swiss-Prot containing computer annotated entries that undergo a number of checks before their publication in UniProtKB/Swiss-Prot.

The data stored in one single file containing the FLAT format records of 20,244 human proteins were obtained from the Expasy portal which is an extensible and integrative portal to access many scientific resources, databases and software tools in different areas of life sciences [5].

We adopted the 1-Letter and 3-Letter standard amino acids abbreviation codes used in UniProtKB/Swiss-Prot, which is the standard adopted by the commission on Biochemical Nomenclature of the IUPAC-IUB [6]. Proteins were labelled according to UniProtKB [4] nomenclature.

## Methods

In UniProtKB/Swiss-Prot, each entry in the FLAT file contains an ID (Identification) line and a SQ (SeQuence header) line with the length of the sequence and the sequence in amino acid. A Perl program was implemented in order to process the data concerning the human proteins contained in the FLAT file format. The output was a table with protein IDs, and the amino acids relative frequencies, which is available at http://hdl.handle.net/2318/836 (or http://aperto.unito.it/handle/2318/836). The program chose each entry in the FLAT file and analyzed the SQ line in order to compute the relative frequencies of each amino acid type in each proteins. The relative frequency was a five digits floating point number with three digits after the decimal point. A hierarchical cluster protein analysis was performed using this table as input for Fastcluster R package [7]. Clustering is the process of partitioning a set of objects into subsets, called cluster, so that each subset contains similar objects, and the objects in separate subsets are dissimilar [8]. The ability of cluster analysis arises from the fact that it can divide similar data without any a priori knowledge. Clustering methods can be divided into two basic types: partitional and hierarchical. A commonly used partitional clustering method is K-means, a process to partition an N-dimensional population into k sets on the basis of a sample [9]. K-means requires that the choice of the number of clusters is made in advance. Given a set of points, a hierarchical clustering creates a binary tree of the data that it successively merges in groups of similar points. Hierarchical cluster only requires a measure of similarity between groups of data points and then it can gradually build clusters. There are two main categories of hierarchical clustering: agglomerative and divisive. An agglomerative clustering starts with a one-point cluster and recursively merges two or more most appropriate clusters. A divisive clustering starts with one cluster of all data points and recursively splits the most appropriate cluster. Agglomerative cluster popularity is largely due to its ability to use arbitrary clustering dissimilarity or distance functions and the conventional wisdom that it produces higher quality trees than divisive or incremental approach [10]. We chose to run the hierarchical cluster analysis for its independence from the choice of the number of clusters. Hierarchical cluster analysis was performed using the R package Fastcluster, which implements fast hierarchical, agglomerative (bottom-up) clustering based on the seven most widely used schemes: single, complete, average, weighted, Ward, centroid and median linkage [7].

### Similarity Measure

Protein sequence clustering is a process which aims to identify sets of homologous proteins in a protein database [11]–[13]. There are many ways to compute similarity between two protein sequences. Generally, the target sequences are aligned depending on the position of the amino acids and the resulting scores are used to calculate a measure of similarity [14].

In our case, the relative frequency of the amino acids in protein sequences was taken as measure of similarity.

The Ward’s method and the Euclidean metric were chosen to compute the distance between the relative frequencies of amino acids in the proteins. The resulting vector of distances was transformed in Newick format using ctc R package [15], in order to be visualized with the graphical editor TreeGraph2 (http://treegraph.bioinfweb.info/) and to extract meaningful subtrees that visualize the distances between clusters.

## Results and Discussion

**Figure 1:**
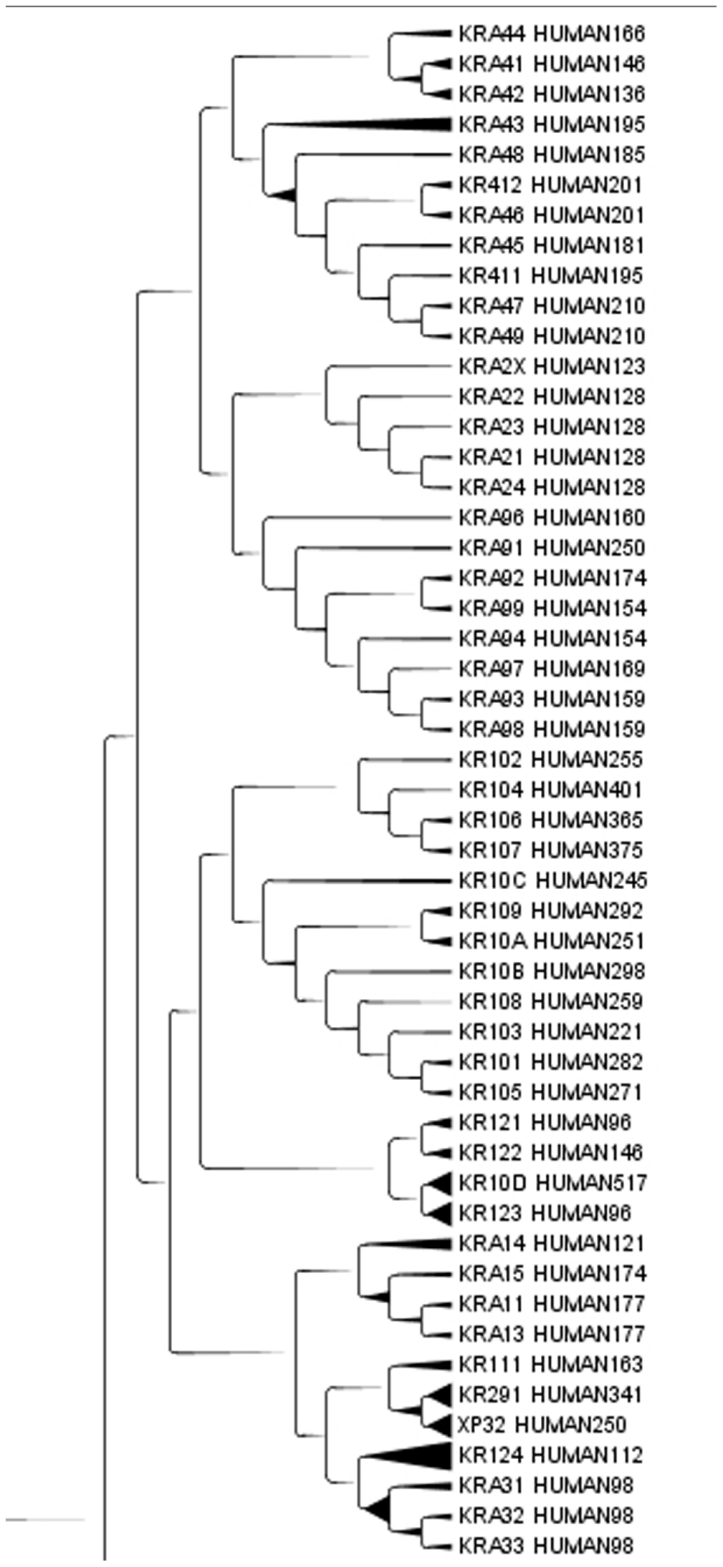
A representative group of Keratin associated proteins. A representative group of keratin associated proteins in the Cladogram from Main Cluster using TreeGraph2. In the Cladrogram we can find KRTAPs family type 1, 2, 3, 4, 9, 10, 11-1, 12, 16, 29-1. The number after HUMAN indicates the length of the protein.

**Figure 2:**
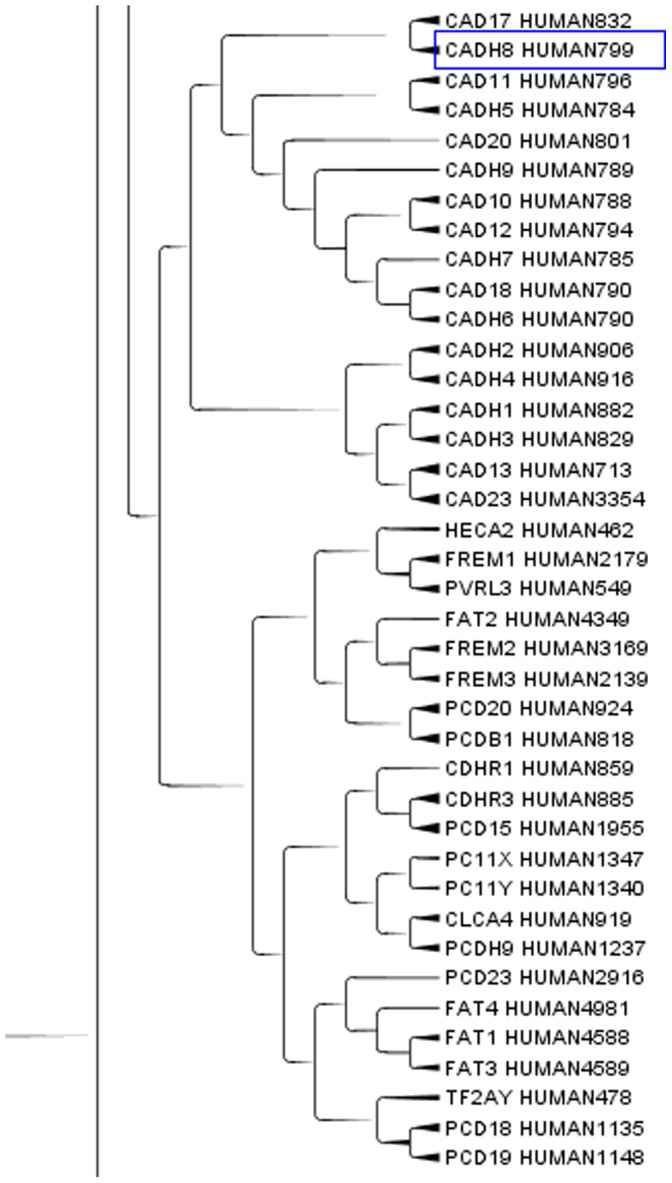
A portion of Cadherins subtree. A portion of cadherin subtree in the Cladogram from Main Cluster using TreeGraph2. In this portion of the Cladrogram we can find the Cadherin domains (5) proteins. The number after HUMAN indicates the length of the protein.

Representation of clusters was given by means of a cladogram. The distribution of proteins along the cladogram was analysed for different groups of proteins belonging to the same group according to sequence similarities. In Figure 1, the cladogram highlights a portion of the group of keratin associated proteins [16]. TreeGraph2 automatically sets line widths or colours according to the value of variables that can be assigned to each node or branch [17]. Keratins are extracellular structural proteins with a very repetitive structure. In this case more than 90% of our clustering overlapped with Swissprot classification. Cadherins, a group of partially extracellular proteins, are another group of proteins that were also distinct from keratin group in cladogram (Figure 2). Figure 2 shows a portion of a cadherin subtree, in which cadherins are mixed with other apparently unrelated proteins.

**Figure 3:**
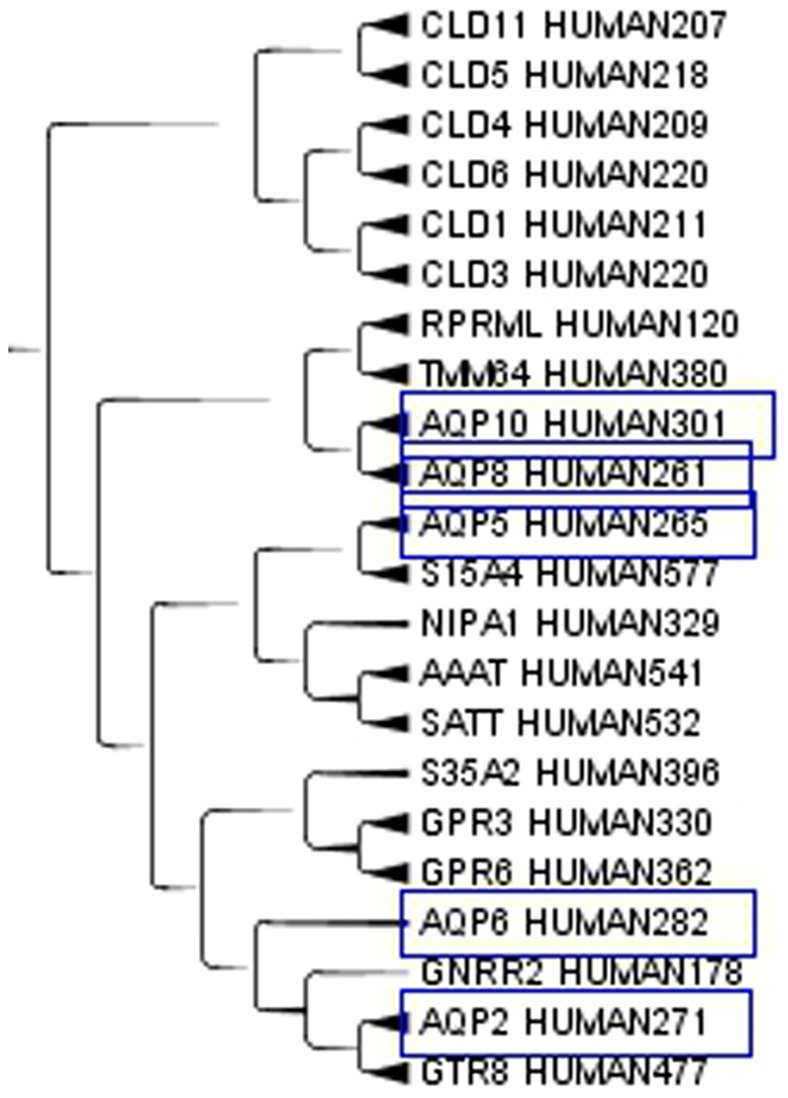
A subset of the Aquaporins. A subset of the Aquaporins from MIP, Major intrinsic proteins Domain, in the Cladogram from Main Cluster using TreeGraph2. The number after HUMAN indicates the length of the protein.

**Figure 4:**
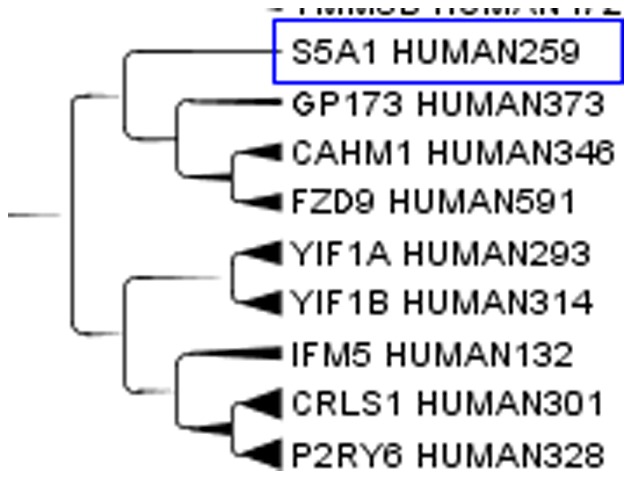
Human steroid 5-alpha reductase, isoform S5A1_HUMAN. Enzyme human steroid 5-alpha reductase, isoform S5A1_HUMAN (SRD5A1 gene) in the Main cluster. The number after HUMAN indicates the length of the protein.

**Figure 5:**
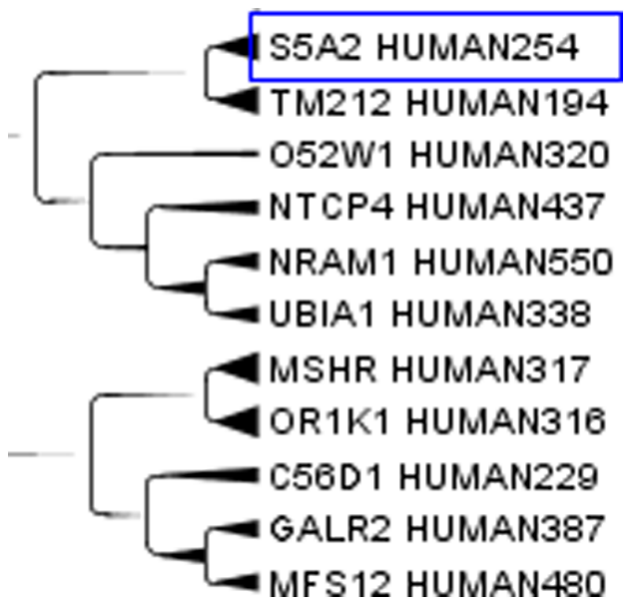
Human steroid 5-alpha reductase, isoform S5A2_HUMAN. Enzyme human steroid 5-alpha reductase, isoform S5A2_HUMAN (SRD5A2 gene) in the Cladogram from Main Cluster using TreeGraph2. The number after HUMAN indicates the length of the protein.

**Figure 6:**
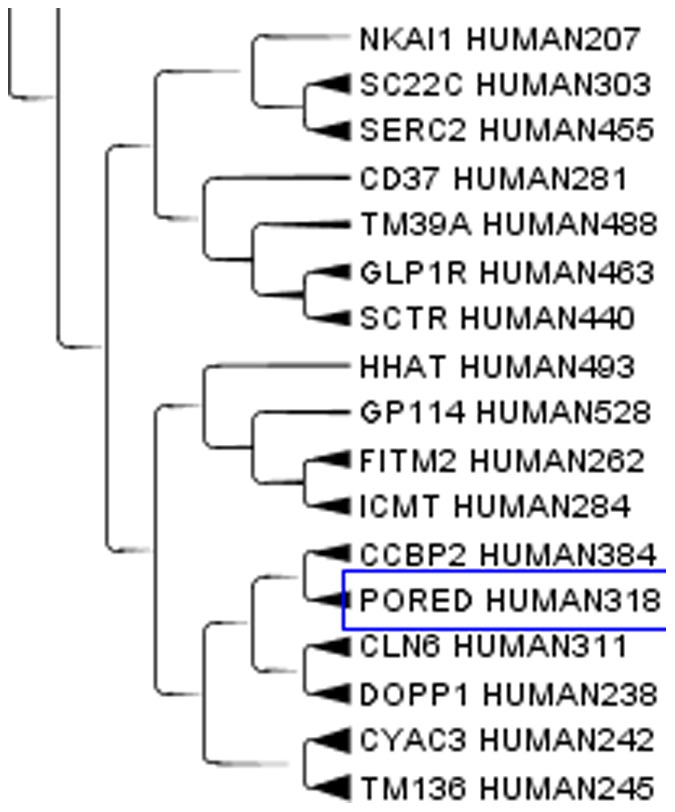
Human steroid 5-alpha reductase, isoform PORED_HUMAN. Enzyme human steroid 5-alphareductase, isoform PORED_HUMAN (SRD5A3 gene) in the Cladogram from Main Cluster using TreeGraph2. The number after HUMAN indicates the length of the protein.

Aquaporins, a set of membrane protein involved in water transport in almost all tissues, see Figure 3, are far apart in the cladogram. This difference means that molecules with a relatively small active site are free to evolve according to the local environment in the moiety less strictly related to the function. Apparently structural proteins have a highly homogenous amino acid composition while catalytic proteins combine highly conserved sites with variable regions that allow clustering according to factors up to now unexplored. When clusterizing only the aquaporins, similarities where observed for those aquaporins on the same chromosome. On the contrary, those on different chromosomes were more distant and they did not clusterize very well. A similar behaviour can be expected for most enzymes that exist in different isoforms. An example of catalytic protein is the enzyme human steroid 5-alpha reductase, that exists in 3 isoforms: S5A1_HUMAN (SRD5A1 gene), S5A2_HUMAN (SRD5A2 gene) and PORED_HUMAN (SRD5A3 gene). They are located on different chromosomes and in our cluster they are not so close, while they are very close to proteins with different functions but similar tissue expression. We analyzed the cluster members of the three isoforms. S5A1_HUMAN, Figure 4, is very close to GP173_HUMAN, CAHM1_HUMAN, FZD9_HUMAN in the cluster. S5A1_HUMAN gene is expressed in foetal brain and ovary, GP173_HUMAN is a super conserved receptor expressed in brain, CAHM1_HUMAN is predominantly expressed in adult brain, FZD9_HUMAN is expressed predominantly in adult and foetal brain. This confirms the closeness in the cluster from the point of view of the tissue. S5A2_HUMAN, Figure 5, is very close to TM212_HUMAN. S5A2_HUMAN is expressed in high levels in the prostate and many other androgen-sensitive tissues, while TM212_HUMAN is a multi-pass membrane protein expressed in the lung. PORED_HUMAN, Figure 6, is very close to CCBP2_HUMAN, DOPP1_HUMAN, CLN6_HUMAN. PORED_HUMAN is expressed in eye, CCBP2_HUMAN in placenta, foetal liver and lung, DOPP1_HUMAN in lung, cerebellum and brain, CLN6_HUMAN in lung and urinary bladder. The apparently inconsistent expression of DOLPP1 in cerebellum and brain may depends on its expression in the glial cells which have a metabolic behaviour more similar to a foetal liver or a lung than to a neuron. This could be a possible explanation for its closeness to CCB2 in the cluster.

### Conclusions

**Figure 7:**
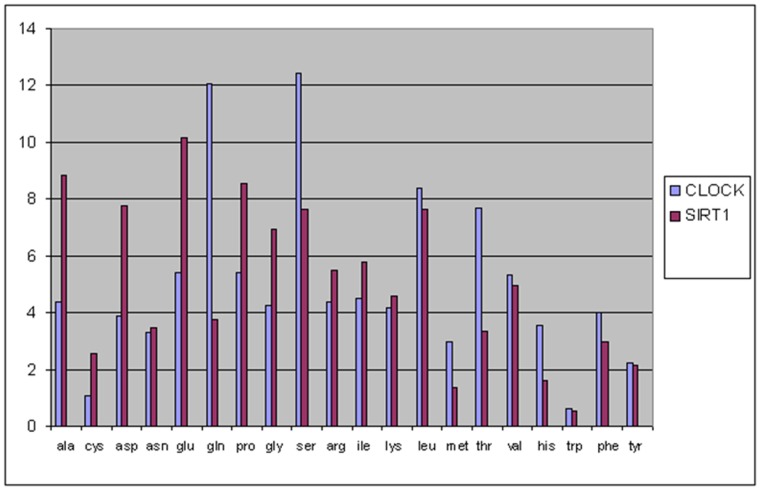
Circadian locomoter output cycles protein kaput and NAD-dependent protein deacetylase sirtuin-1 amino acids relative frequencies. The graph compare the relative frequencies of the Circadian locomoter output cycles protein kaput (CLOCK gene) and NAD-dependent protein deacetylase sirtuin-1 (SIRT1) highlighting the different level of glutamate and glutamine. CLOCK means high glutamine and bases synthesis and switch on DNA synthesis. SIRT1 means low glutamine and high glutamate and acetylCoA and switch off DNA synthesis.

Proteins with a repetitive structure and with highly specific AA patterns such as keratins and collagens cluster quite well demonstrating the correctness of the mathematical approach, but their clustering added no information to existing knowledge. Proteins that clusterize on the basis of AA percentage but perform quite different functions or similar functions in different tissues or microenvironments (glial cells and neurons in the same area have a completely different glutamate/glutamine) disclose new approaches to the description of complex biological systems. Polymorphic proteins performing similar functions in different tissues (e.g. highly oxygenated/hypoxic) have different AA percentages which allow more efficient protein synthesis, and so on. The cell cycle is a cyclic process alternating DNA and protein synthesis. DNA synthesis requires a high amount of glutamine. While protein synthesis relies on the presence of all AA. Clock and SIRT1 proteins [18], (Figure 7), are widely accepted as regulator check point of cell cycle. Considering the glutamine content, CLOCK should be higher during DNA synthesis while SIRT1 during protein synthesis. This has been well known for years, merely on the basis of experimental data. Now we can understand why: protein AA content depends on local AA content and becomes a “signal” that activates the proper metabolic pathway. AA percentage becomes a relevant part of the “information content” of the protein.

In conclusion, this method makes it possible to gather so far unexplored information on proteins, linking their coordinated expression to chromosome or tissue locations, cell cycle phase, starvation and other metabolic constraints. It is potentially very useful for predictive analysis before passing on to expensive and time-consuming laboratory tests.
